# Antioxidant, Physiochemical, and Sensory Properties of Functional Marshmallow Produced from Honey, Strawberry Concentrates, and Hibiscus Extract

**DOI:** 10.3390/foods14020265

**Published:** 2025-01-15

**Authors:** Maher M. Al-Dabbas, Etaf G. Abu Samaan, Sehar Iqbal, Hani J. Hamad, Rawan Al-Jaloudi, Mohammad Shahein, Bha’a Aldin Al-Nawasrah, Abdalrahman Al-Zabt, Doa`a Al-Refaie, Nisreen Shehadeh, Mahmoud Abughoush

**Affiliations:** 1 Department of Nutrition and Food Technology, Faculty of Agriculture, The University of Jordan, Amman 11942, Jordan; m.aldabbas@ju.edu.jo (M.M.A.-D.); etaf.gh@hotmail.com (E.G.A.S.); nisreen_shehadeh@yahoo.com (N.S.); 2 Nutrition and Dietetics Program, College of Pharmacy, Al Ain University, Abu Dhabi 112612, United Arab Emirates; mahmoud.abughoush@aau.ac.ae; 3 Department of Clinical Nutrition and Dietetics, Faculty of Allied Medical Sciences, Philadelphia University, Amman 19392, Jordan; hhamad@philadelphia.edu.jo; 4 Department of Allied Medical Sciences, Zarqa University College, Al-Balqa Applied University, Zarqa 13110, Jordan; rawan.aljaludi@bau.edu.jo (R.A.-J.); mohammad.shaheen@bau.edu.jo (M.S.); 5 Nabeel Ghunaim and Partners Company, Amman 11181, Jordan; dr.bhaa.alnawasrah@icloud.com; 6 Border Inspection Section, Food Safety Department, Ministry of Public Health, Doha 774, Qatar; alzabt995@gmail.com; 7 School of Hospitality and Tourism, Luminus Technical University College, Amman 11118, Jordan; 8 Department of Clinical Nutrition and Dietetics, Faculty of Applied Medical Sciences, The Hashemite University, Zarqa 13133, Jordan

**Keywords:** DPPH, flavonoid, phenolic content, reducing power, texture analysis, marshmallow

## Abstract

Marshmallow candy is a well-known sugar-based confection that is widely consumed among different population groups. However, its high sugar contents and low nutritive value might lead to adverse health outcomes. This study, therefore, aimed to develop optimal formulations for functional marshmallow candy using honey, aqueous hibiscus extract, cow’s milk, and strawberry concentrates with partial replacement of table sugar and glucose syrup. In this regard, six different formulations (F1–F6) were developed, varying in the ratios of key ingredients, such as F1 formulated with honey (9%), sucrose (35%), glucose syrup (33%), milk (4%), and hibiscus extract (1%); F2 containing honey (10%), sucrose (30%), glucose syrup (25%), milk (4%), hibiscus extract (1%), and strawberry concentrate (13%); F3 with honey (15%), sucrose (25%), glucose syrup (20%), milk (4%), hibiscus extract (1%), and strawberry concentrate (16%); F4 formulated with honey (20%), sucrose (20%), glucose syrup (15%), milk (4%), hibiscus extract (1%), and strawberry concentrate (24%); F5 containing honey (30%), sucrose (20%), glucose syrup (5%), milk (4%), hibiscus extract (1%), and strawberry concentrate (24%); and F6 developed with honey (10%), sucrose (30%), glucose syrup (25%), and strawberry concentrate (16%) without milk or Hibiscus extract. These formulations were evaluated for total phenolic content, total flavonoid content, DPPH radical scavenging activity, and textural and sensory characteristics. The results show that all formulations containing functional ingredients had significantly higher total phenolic and flavonoid contents, along with stronger antioxidant activities in a dose-dependent manner compared to the control. Among the formulations, F5 exhibited the highest phenolic and flavonoids contents (89.8 mg GAE/100 g and 1.62 mg RE/100 g, respectively) and reducing power activity (197.8% equivalent to 30 µg vitamin C). Additionally, F3 and F4 showed the strongest DPPH scavenging activity, with IC_50_ values of 8.0 and 5.1 mg/mL, respectively. In terms of texture, the hardness of all formulations was comparable to the control, except for F1 and F6. The results for sensory analysis show that all modified marshmallows received higher consumer acceptance in overall liking, appearance, softness, elasticity, lightness, and flavor compared to the control. Overall, this study shows that the use of functional ingredients significantly enhanced the total phenolic and flavonoid content and improved antioxidant activities in marshmallow production. This functional candy can provide nutrient-rich health-promoting ingredients for consumers.

## 1. Introduction

Confection products, including sweets, candies, toffees, marshmallows, jelly, chewing gum, and chocolates, are widely consumed around the world. Different age groups, particularly children, are more captivated by their radiant colors, shapes, flavor, and textural properties [1]. It is estimated that one-third of United States (US) children consume candy with an average of 9.84 kg/capita/day, and more than 7 billion pounds of candies are manufactured each year in the country [2]. Despite their huge production, confectionery products are packed with added sugars, food additives, and synthetic colors and flavors, making them less nutritive and caloric-dense foods [3]. Previously, many studies have shown a strong association between high sugar intake and several adverse health outcomes including obesity, diabetes [4], cardiovascular diseases [5], dental caries [6], and even risk of cancer [7]. Therefore, the World Health Organization (WHO) recommended that limiting free sugars to less than 10% of total energy intake for both adults and children can help to minimize the related health risks [8]. Equally, consumers’ awareness and demand for nutritious food has been increased considering the high prevalence of chronic diseases [9].

The enrichment of sugar confectionery with functional ingredients in this regard would supplement the diet with biologically active nutritional substances to improve overall functional properties and more consumers acceptability [10]. Functional foods and nutraceuticals can provide traditional nutrients and health benefits for disease prevention. For example, hibiscus flowers are rich in phytochemicals, antioxidants, fibers, essential fatty acids, aldehydes, flavonoids, prebiotics, and probiotics [11]. They contain natural food colorant, cyanidin diglucoside, flavonoids, and a wide range of vitamins including thiamine, riboflavin, niacin, and ascorbic acid [12]. Similarly, fruits are an excellent source of nutrients, and phytochemicals help to reduce the risk of major chronic diseases [13]. For example, strawberries belong to the berry group and are low in calories and rich in vitamin C, fiber, pectin, and numerous polyphenolic compounds [14]. Milk contains various components with numerous functional benefits high in protein, ash, calcium, and vitamins A and C [15], while natural honey is rich in vitamins, phenolic compounds, organic acids, amino acids, and other essential minerals. Through its anti-inflammatory and antimicrobial properties, honey helps to improve the antioxidant capacity, lipid profile, regulation of glycemic responses, and modulation of the immune system [16].

Considering that, sugar and corn syrup can be replaced, either completely or partially, within recipes with honey and/or fruit concentrates to produce functional candies such as marshmallows [17]. Marshmallow is a non-crystalline confectionary product with an elastic and foamy structure developed through the addition of gelling and aerating agents in a boiled sugar mixture [18]. Marshmallows are produced mainly by cooking equal parts of sucrose and glucose syrup to 80% Brix at 110 °C, followed by the addition of dissolved gelatine solution, colors and artificial flavors [19]. Thus, the use of natural fruit concentrates, milk, natural honey, and aqueous hibiscus extract in marshmallow candy can substitute the part of the table sugar by improving its nutritional value and reducing the risk of diseases. However, in reformulating confection products by replacing sugar with nutritive-rich ingredients, it is a huge challenge for the manufacturers to ensure consumer satisfaction and recommendation. Therefore, the objective of this study was to find the best formulation to produce functional marshmallow candy from honey, fruit concentrates, milk, and hibiscus extract to provide a substitute for traditional table sugar and glucose syrup. Further, the antioxidant activities and total phenolic and flavonoid contents of the modified marshmallow were analyzed to measure the nutritional value of the new product.

## 2. Materials and Methods

### 2.1. Ingredients of Marshmallows

To produce these marshmallows, sucrose, glucose syrup (Aknisasta, Istanbul, Turkey), gelatin 220 Bloom value (Gelita, Cotia, Brazil), red color (Rouge food’s color, Valetta, Malta), mango essence flavor (Foster Clark Products Ltd., Valetta, Malta), honey (Langnese, Bargteheide, Germany), strawberry concentrate (Pregel S.p.a, Frazione Arceto, Italy), and hibiscus were purchased from local market (Alalla, Amman, Jordan).

### 2.2. Preparation of Marshmallow Confection

Marshmallows are highly non-crystalline aerated sugar syrup. Therefore, gelatin was used as the whipping and setting agent. Traditional (control) marshmallow was prepared by cooking equal parts of sucrose (35%) and glucose syrup (35%) to 110 °C Brix (80%), followed by the addition of dissolved gelatin solution (4%) that was previously heated to 60 °C. After cooling (29–35 °C), the mixture was whipped with coloring and flavoring materials with a mechanical agitator (KENWOOD, Prospero KM242, Solihull, UK) followed by a gradual increase in speed to achieve maximum yield. The final foamy product was placed into a warm dry starch bed for 24 h at room temperature. The previous production steps were followed to produce the modified marshmallows using different functional products, as shown in Table 1, using the mixture response surface experimental design. The produced marshmallows from each treatment were taken for sensory, chemical, and textural analysis.

### 2.3. Nutritional Value and Cost of Modified Marshmallows

The nutritive value of the modified marshmallows was calculated separately considering the proportion of ingredients used in different marshmallows formulations. The nutritive value was calculated using the USDA tables for food composition [20]. Specifically, the content of individual components (e.g., protein, carbohydrates, fats, vitamins, and minerals) in each ingredient was obtained from the USDA database, and their respective contributions to the final product were determined based on the ingredient ratios used in the formulations. The economy of marshmallows production cost (USD/100 g) was determined through calculating the cost of fraction used from the main raw materials (local market unit price) according to the different treatments/formulations of marshmallows.

### 2.4. Chemical Analysis

#### 2.4.1. Chemicals

Gallic acid, rutin, aluminum trichloride (AlCl_3_), ferric chloride (FeCl_3_), and L-ascorbic acid were purchased from sigma-aldrich (Steinheim, Germany). Folin–Ciocalteu reagent and trichloroacetic acid were purchased from AppliChem GmbH (Darmstadt, Germany). 1,1-diphenyl-2-picrylhydazyl (DPPH) was purchased from ICN Biomedicals Inc. (South Chillicothe Road, Aurora, OH, USA). Di-Sodium hydrogen phosphate extra pure (Na_2_HPO_4_.2H_2_O), sodium carbonate (Na_2_CO_3_), and potassium ferricyanide (K_3_[Fe(CN)_6_]_6_) were purchased from E. Merck (Darmstadt, Germany). Sodium dihydrogen phosphate (NaH_2_PO_4_.2H_2_O) was purchased from Fluka-Garantie (Buchs, Switzerland). Sodium hydroxide (NaOH, HPLC grade) was purchased from LABCHEM laboratory chemicals (Zelienople, PA, USA). Other chemicals were of reagent grade and purchased from local companies.

#### 2.4.2. Sample Preparation for Analysis

In order to analyze the sample, the aqueous hibiscus infusion was prepared by soaking 10.0 g of dried hibiscus herbs in pre-boiled water for 5 min, and the filtrate was diluted to 100 mL. The total phenolic and flavonoid contents, DPPH free radical scavenging and reducing power activities, and water extract 10% (*w*/*v*) from the produced marshmallows were determined. Honey and strawberry concentrate were prepared separately by dissolving an equivalent amount of 10.0 g (FWB) in 100 mL distilled water.

#### 2.4.3. Moisture Content

The moisture content (%) of the final products was determined in duplicate using a conventional oven at 105 °C until constant weight was achieved (Memmert, Model UFE500, Schwabach, Germany).

#### 2.4.4. Determination of Total Phenolic Content

The total phenolic content present in honey, hibiscus extract, strawberry concentrate, and produced marshmallows was determined separately using a Folin–Ciocalteau reagent with slight modification [21]. In brief, 0.5 mL of each sample (10 g/100 mL) was transferred into a 10 mL volumetric flask, followed by the addition of 2.5 mL of distilled water. The Folin–Ciocalteu reagent (250 µL) was then added and mixed thoroughly. After 3 min, 0.5 mL of 10% sodium carbonate (10 g/100 mL) was added, and the absorbance was measured at 760 nm with a spectrophotometer (model UVD-2900, Labomed, Los Angeles, CA, USA). Gallic acid was used as the standard for a calibration curve. The total phenolic compound contents (mg/100 g) were expressed as the Gallic acid equivalent (GAE) and determined from the following regression equation based on the established calibration curve:

Y = 0.074X,    R^2^ = 0.996

where Y is the absorbance, and X, the Gallic acid concentration in mg/L. All measurements were carried out in triplicate.

#### 2.4.5. Determination of Total Flavonoid Content

The content of flavonoids was determined using the Miliuskas method with slight modification [22]. An aliquot of 0.5 mL from each treatment was mixed with 1 mL of 2% aluminum trichloride in ethanol solution and diluted with water into 25 mL. The absorption at 415 nm was taken after 40 min at 20 °C (Labomed spectrophotometer, model UVD-2900, Labomed, Los Angeles, CA, USA). The total flavonoid contents (mg/100 g) in honey, hibiscus extract, strawberry concentrate, and modified marshmallows were expressed as the Rutin equivalent (RE) and determined from the following regression equation based on the established calibration curve:

Y = 1.194X + 0.0059,    R^2^ = 0.996

where Y is the absorbance, and X, the Rutin concentration in mg/L. All measurements were carried out in triplicate.

### 2.5. Determination of Antioxidant Activities

#### 2.5.1. DPPH Free Radical Scavenging Assay

DPPH (1,1-diphenyl-2-picrylhydrazyl) was used to determine the free radical scavenging activity in honey, hibiscus extract, strawberry concentrate, and produced marshmallows using the method of Hatano with some modification [23]. Briefly, 0.1, 0.5, 0.75, and 1.0 mL from each treatment previously dissolved in water (10 g/l00 mL) was mixed with 1 mL of a methanolic solution of DPPH (6 × 10^−5^ M), and the mixture was mixed by Vortex. The absorbance of each treatment was measured at 517 nm (Labomed spectrophotometer, model UVD-2900, Labomed, Los Angeles, CA, USA) after 30 min against a blank. The IC50 value (the concentration required to inhibit 50% of DPPH) for each treatment was calculated from a dose–response curve between DPPH inhibition % and concentrations.

$$\mathrm{DPPH}\;\mathrm{free}\;\mathrm{radical}\;\mathrm{inhibition}\;\mathrm{activity}\;(\%)\;=\;\frac{\mathrm{Control}\;\mathrm{absorbance}\;-\;(\mathrm{Sample}\;\mathrm{absorbance}\;-\;\mathrm{Blank}\;\mathrm{absorbance})}{\mathrm{Control}\;\mathrm{absorbance}}\;\times \;100$$

#### 2.5.2. Reducing Power Assay

The reducing powers of honey, hibiscus extract, strawberry concentrate, and produced marshmallows were determined using the Yildirm method with some modification [24]. Briefly, 0.2 mL from each treatment previously dissolved in water (10 g/100 mL) was mixed with 2.5 mL phosphate buffer (0.2 M, pH 6.6) and 2.5 mL potassium ferricyanide (1 g/100 mL). The mixture was then incubated at 50 °C for 30 min followed by the addition of 2.5 mL trichloroacetic acid (10 g/100 mL) and centrifugation at 1650× *g* for 10 min. After that 2.5 mL of upper layer solution was taken and mixed with 2.5 mL ferric chloride (0.1 g/100 mL). The absorbance was measured at 700 nm for treatments and the standard of 30 µg ascorbic acid (0.1 g/100 mL).

$$\mathrm{Antioxidant}\;\mathrm{activity}\;(\%)\;=\;\frac{\mathrm{Sample}\;\mathrm{absorbance}}{\mathrm{Ascorbic}\;\mathrm{acid}\;\mathrm{absorbance}}\;\times \;100$$

### 2.6. Textural Analysis

The produced marshmallow hardness was determined using a Texture analyzer (Multitest 1-i, Mecmesin, Slinfold, UK) fitted with a 10 mm probe. The test speed was 0.5 mm/s, and the probe was allowed to progress 10 mm into each sample. The maximum force in newton (N) was used as an expression of marshmallow hardness. All treatments were conducted in triplicate.

### 2.7. Sensory Analysis

A sensory evaluation of the control and modified marshmallows was conducted in the Food Science Laboratories at the University of Jordan. Consumers (31 students and staff) who were familiar with the quality characteristics of marshmallows were selected to quantify the following quality attributes: overall acceptability, appearance, softness, lightness, flavor, and elasticity. A 9-point hedonic scale (1 = dislike extremely to 9 = like extremely), according to Meilgaard et al., was used to rate the quality attributes for each sample [25]. All samples were coded using random three-digit numbers in a randomized serving order, and all samples were evaluated in duplicate.

### 2.8. Statistical Analysis

Statistical calculations were performed using statistical analysis system, SAS program, 2000 (SAS Institute Inc., Cary, NC, USA). Significant differences among the means of the treatments were determined using an LSD test. Differences at *p* < 0.05 were considered significant. Regression equations and correlation coefficients (R) were determined with MS Excel software. All treatments were conducted in triplicate.

## 3. Results

### 3.1. Moisture Contents and Texture Analysis

The results presented in Table 2 show the average moisture contents and texture analysis of the produced marshmallows with different formulations. The moisture content ranged from 11.9 to 21.1%, where the moisture content of the control marshmallow was higher (21.1%) compared to that of the other treatments, while no significant differences (*p* < 0.05) were observed among the different modified marshmallows (F1–F6).

For the texture analysis, the force in N needed to move 10 mm through the produced marshmallows ranged from 0.62 to 1.17 N. The higher the force needed to move through the modified marshmallows, the harder or less soft the texture. In this regard, the F1 treatment was shown to have highest hardness value (1.17 ± 0.13 N), while F6 reported the least hardness (0.62 ± 0.11). Further, the decreasing order of the hardness values of all formulations, i.e., F1 > F5 > F3 = Control > F4 > F2 > F6, is shown in Table 2.

The traveled distance (mm) of the probe through the marshmallow samples at 0.5 N of applied shear force was obtained from stress–strain curves. The F1 treatment reported the least traveled distances (2.61 ± 0.92c mm), while F6 showed a value of (6.05 ± 0.73a mm). Both were significantly different (*p* < 0.05) from the control and other treatments, while the F2, F3, T4, and F5 treatments were shown to have a hardness similar to that of the control group with no significant difference.

### 3.2. Total Phenolic and Flavonoid Contents of the Modified Marshmallows

The average total phenolic compound contents for all produced marshmallows are shown in Table 3. The total phenolic content of the produced marshmallow samples (F1–F6) ranged from 44.6 ± 5.3 mg to 89.8 ± 4.7 mg GAE/100 g. In addition, the phenolic contents of the produced marshmallow using functional ingredients from honey, strawberry concentrate, and hibiscus extract were significantly different (*p* < 0.05) from those of the control (14.0 mg GAE/100 g).

Our results show that the flavonoid content for the modified marshmallow treatments was significantly different (*p* < 0.05) from that of the control. The averages of the total flavonoid contents for the produced marshmallows varied from 0.03 mg RE/100 g in the control to 1.62 mg RE/100 g in F5 (Table 3).

#### 3.2.1. DPPH Inhibition Activity of the Modified Marshmallows

Regarding DPPH radical scavenging activity, the IC_50_ (the concentration required to inhibit 50% of DPPH free radicals) for the ingredients and modified marshmallows is also presented in Table 3. Based on our results, IC50 values ranged from 99.6 to 5 mg/mL, where the IC50 value for the control was the highest (99.6 ± 1.9 mg/mL), whilst F4 showed the lowest IC50 value (5 ± 3.1 mg/mL). Furthermore, the IC50 values of strawberry concentrate, hibiscus extract, and honey were observed at 4.10 ± 3.3, 50.9± 2.6, and 72.1 ± 1.8 mg/mL, respectively.

**Figure 1:**
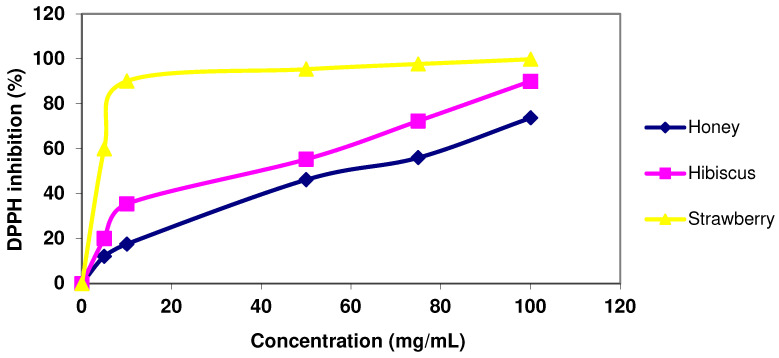
DPPH radical scavenging activity of honey, hibiscus extract, and strawberry concentrate at different concentrations.

**Figure 2:**
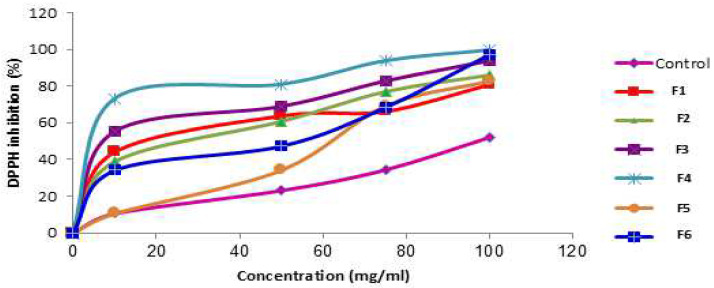
DPPH radical scavenging activity of all marshmallow treatments at different concentrations.

The quenching of DPPH radical color with the modified marshmallows and/or ingredients used to produce them was in a concentration-dependent manner (Figure 1 and Figure 2). The strawberry concentrate showed complete color inhibition at concentrations of 50, 75, and 100 mg/mL due to its high phenolic and flavonoid content (Figure 1), while all of the treatments and/or ingredients were significantly different (*p* < 0.05) from the control (Figure 2). The DPPH radical scavenging activity at the concentration of 75 mg/mL for all treatments showed a strong correlation of R = 0.84 with the content of phenolic compounds, and R = 0.72 with the total flavonoid content.

#### 3.2.2. Reducing Power Activity of the Modified Marshmallows

**Figure 3:**
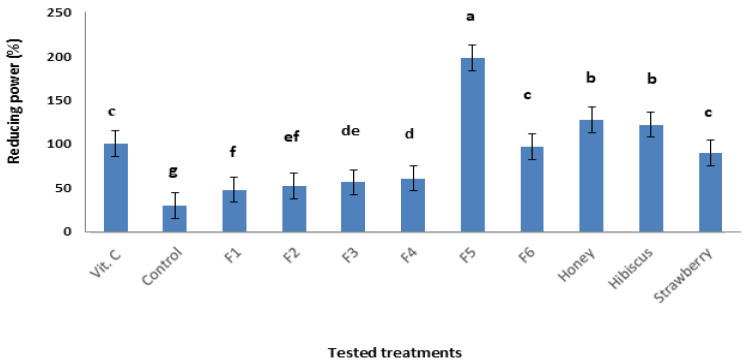
Reducing power (%) of modified marshmallows (formulation 1–6), standard vitamin c, honey, hibiscus, strawberry and control. Means followed by different letters are significantly different (*p* < 0.05).

The reducing power results ranged between 29.4 and 197.8% as 30 µg of vitamin c equivalents at a tested concentration of 20 mg from each treatment. The reducing power results (Table 3) show that the F5 treatment possesses the highest reducing power activity (197.8 ± 4.0) among all treatments. Additionally, Figure 3 presents the comparison between the reducing power activities of treatments and the standard ascorbic acid (30 µg). The control treatment shows the lowest reducing power activity, indicating that the control had very weak antioxidant activity compared to ascorbic acid. The results of reducing power are in the following decreasing order: F5 > F6 > F4 > F3 > F2 > F1 > control. The reducing power (%) for all treatments shows a correlation of R = 0.45 with the phenolic content, and R = 0.44 with flavonoid content.

#### 3.2.3. Sensory Analysis

From the sensory analysis, the highest softness score was observed for F6 (6.87), and the lowest was reported for F2 (5.90) (Table 4). Similarly, textural analyses showed that F6 was the softest, whilst F2 was the hardest in texture among the other treatments evaluated by consumer’s feedback. Moreover, T4 had the highest average scores in overall acceptability (6.96), appearance (7.13), and lightness (6.80) and showed the most preferred soft texture and ingredient concentration. In addition, T2 had the lowest averages in appearance (5.65), softness (5.90), lightness (5.61), elasticity (5.65), and flavor (5.03) and was significantly different from all the other treatments. The maximum elasticity score was 6.93 for T5, the lowest was 5.65 for T2, and both were statistically different from the control.

#### 3.2.4. Production Cost and Nutritional Value of the Modified Marshmallows

The nutritive value and economy of the traditional and modified marshmallows were calculated separately with respect to the proportion of added ingredients (Table 5). The cost of each fraction of honey, strawberry concentrate, sugar, hibiscus extract, glucose syrup, and milk used in the treatments was determined, and the total recipe cost for producing each formulation was calculated. Additionally, the final product cost in US dollars (USD/100 g) was estimated.

## 4. Discussion

Our results show that all marshmallow formulations containing functional ingredients showed high antioxidant activities and total phenolic and flavonoid contents compared to the control group. Similarly, the modified marshmallows will have a longer shelf-life than control due to the reduced moisture content while comparing to control group. The moisture content of the control marshmallow was higher in our study than with other treatments, while no significant differences (*p* < 0.05) were observed among the different modified marshmallows. This can be attributed to the increase in the concentration of the total soluble solids from the addition of new ingredients to the modified marshmallows [26]. Water activity is an important parameter for confectionery products that determines microbiological activities, storage conditions, and chemical stability. Hence, bacterial growth is inhibited below water activity of 0.85, while the growth of molds and yeasts is considerably slowed at approximately 0.70, with no growth occurring below water activity of 0.60 [27]. In addition, modified marshmallows showed high nutritive value when replacing the glucose or sugar syrup with honey, milk, and strawberry concentrates. Similarly to our findings, Ali and colleagues developed jelly candy from fruit extracts and reported that the use of fruit mixtures could improve candy functionality, nutritional value, and flavor [28]. Similarly, Tamer et al. (2013) showed that fortified jelly with a mixture of vitamins and minerals from apple concentrate, natural flavor, and color could improve the nutritional contents of jelly [29]. Another study reported that strawberry jam and hard candy fortified with hibiscus at different concentrations tend to increase the functional benefit of the product due to high flavonoid content, rich pigments, and wide range of antioxidant vitamins [30].

Our results show that use of functional ingredients at different concentrations subsequently raises the phenolic content for different formulations. Polyphenols are naturally occurring, non-nutritive compounds existing in fruits, vegetables, herbs, and plants [31,32]. Phenolic and flavonoid compounds have robust antioxidant activity and help to prevent degenerative diseases. Studies have shown that honey, strawberries, and hibiscus are rich in phenolic contents. For example, earlier studies found that the phenolic content of different types of honey ranged from 25.7 to 67.9 mg GAE/kg [33,34]. Similarly, the highest value of total phenolic content of 273 mg GAE/100 g was found in strawberries [35]. In addition to phenolic value, the flavonoid content for the modified marshmallow treatments with functional ingredients was significantly higher than that of the control. It was reported that the fortification of candy with functional ingredients increases their phenolic and antioxidant activities [29]. Previous studies showed that honey, hibiscus, and strawberry contain different amounts of flavonoid contents. Honey’s flavonoid content range was reported at 0.17–8.35 mg Quercetin equivalent (QE/100 g) [36], strawberry varied from 70.5 to 46.2 mg catechin equivalent (CE/100 g) [35], and hibiscus’ total flavonoid content was observed at 1.6 mg CE/100 g [37].

Regarding the IC_50_ (the concentration required to inhibit 50% of DPPH free radicals), it is important to note that the lower the IC_50_ value of the treatments, the higher its antioxidant activity. Antioxidants are a very wide group of compounds that can scavenge certain radicals. Total antioxidant activity assays usually measure the ability of these compounds to retard lipid peroxidation or chelating metal ions [38]. In this regard, the DPPH inhibition technique is a concentration-dependent assay; as the concentration of total antioxidant activity increases, more DPPH free radical scavenging activity will occur, and lower values will be obtained. This action is accompanied by decolorization, which is an indicator for DPPH quenching [39]. The IC_50_ value for the control was the highest among the treatments, while F4 showed the lowest IC_50_ value. All modified marshmallows showed a low IC_50_ value compared to that of the control group due to added ingredients. In agreement with our results, Vanker and Srivastava (2008) suggested that red hibiscus has very strong DPPH inhibition (%) due to its content of anthocyanins and vitamin c [37]. Additionally, strawberries contain 540 mg/kg ascorbic acid, which is known to be a robust antioxidant [40].

Reducing antioxidant power activity measures the ability of a substance to reduce Fe^3+^ to Fe^2+^ to analyze the antioxidant activity of any substances [41]. Our results show that the F5 treatment possess the highest reducing power activity among all treatments, while the control showed the lowest reducing power activity, indicating that the control had a very weak antioxidant activity. Previously, it was suggested that the total antioxidant activity of strawberry’s reducing power range was 36.7–134.1 µmol/g (vitamin c equivalent) [35]. Furthermore, another study reported a total antioxidant activity of 6.8% TE for red hibiscus [37].

For texture and sensory analysis, all modified marshmallows received higher consumer acceptance in overall liking, appearance, softness, elasticity, lightness, and flavor compared to the control. These findings indicate that the increases in strawberry concentrate, honey, glucose syrup, and sucrose levels in marshmallow production can provide a better texture. This may be attributed to the strength of the new texture as a result of mixing a high percentage of sucrose and honey with glucose syrup, similarly to the control. Also, marshmallow hardness can be affected by ingredient modification; for example, Walton et al. found that milk and honey mixtures increased foam-like candies’ stability and hardness [42]. Correspondingly, another former study found that the hardness of fortified marshmallow with natural pigments was increased in comparison to that of unfortified products [43]. Furthermore, the hardness of marshmallow samples produced from different combinations of isomaltulose, fructose, and glucose syrup instead of sucrose increases in value [44]. Flavor scores showed significant differences between all treatments compared to the control. More specifically, F1, F3, and F4 were not significantly different (*p* < 0.05) among the different formulations. The results might be due to similarities in the concentrations of the used ingredients and the same organoleptic qualities. Our results are similar to the findings of a previous study that found that consumers’ degree of liking modified marshmallows increased with the increase in bee pollen concentrations [10].

### Study Limitations

This study shows that marshmallows containing functional ingredients have high antioxidant activities and total phenolic and flavonoid contents and might have more health benefits; however, our study has a few limitations. For example, the nutritive value of all formulations was estimated according to the percentage used from each ingredient according to the labels or USDA tables. Since the main idea was to show the improvement in antioxidant activity by measuring certain parameters (phenolic and flavonoid contents), the calculation of the nutritive value was not an aim of this study, and this can be considered a study limitation. Similarly, this study focused on determining the moisture content of the samples while water activity was not measured in this study. However, future work could include measuring water activity to further assess the microbial and storage stability of the formulations.

## 5. Conclusions

Our results indicate that the use of functional ingredients (cow’s milk, honey, hibiscus extract, and strawberry concentrate) increases the antioxidant activity and total phenolic and flavonoid contents of the modified marshmallows compared to the control marshmallow. Similarly, the modified marshmallows reported higher reducing power compared to the control group, indicating higher functional properties. Based on our findings, this functional candy might help to provide nutrient-rich health-promoting ingredients for consumers and manufacturers.

## Figures and Tables

**Table 1:** Composition and functional ingredients of modified marshmallows.

Trials	Gelatin (%)	Water (%)	Glucose Syrup (%)	Honey (%)	Milk (%)	Sugar (Sucrose) (%)	Hibiscus Extract (%)	Strawberry Concentrate, 60 Brix (%)
Control	4	25	35	-	-	35	-	-
F1 *	4	14	33	9	4	35	1	-
F2	4	13	25	10	4	30	1	13
F3	4	15	20	15	4	25	1	16
F4	4	12	15	20	4	20	1	24
F5	4	12	5	30	4	20	1	24
F6	4	15	25	10	-	30	-	16

F *: formulations of modified marshmallow; *n* = 1, 2, 3, 4, 5, and 6.

**Table 2:** Average moisture content, hardness, and traveled distance of different marshmallow formulations.

Formulation *	Moisture Content (%)	Hardness (N)	Traveled Distance at 0.5 N (mm)
Control	21.1 ± 2.2 ^a^	0.90 ± 0.05 ^bc^	5.15 ± 0.34 ^ab^
F1	11.9 ± 1.8 ^b^	1.17 ± 0.13 ^a^	2.61 ± 0.92 ^c^
F2	13.4 ± 1.9 ^b^	0.80 ± 0.08 ^cd^	4.65 ± 0.62 ^b^
F3	12.2 ± 2.5 ^b^	0.90 ± 0.09 ^b^	4.27 ± 0.46 ^b^
F4	13.8 ± 3.2 ^b^	0.88 ± 0.17 ^bc^	4.48 ± 0.73 ^b^
F5	14.2 ± 1.9 ^b^	0.95 ± 0.30 ^bc^	4.48 ± 1.12 ^b^
F6	12.9 ± 1.6 ^b^	0.62 ± 0.11 ^d^	6.05 ± 0.73 ^a^

* Results are means ± standard deviation of triplicate analysis. Means in the same columns followed by different letters are significantly different (*p* < 0.05).

**Table 3:** DPPH inhibition activity, reducing power, and phenolic and flavonoid content of the modified marshmallows.

Treatments	Phenolic Content as Gallic Acid (mgGAE/100 g)	Flavonoid Content as Rutin (mg RE/100 g)	IC_50_ Values for DPPH Inhibition (mg/mL)	Reducing Power (%) (30 µg Vitamin C Equivalent)
Control	14.0 ± 1.8 ^f^	0.03 ± 0.01 ^f^	99.6 ± 1.9 ^a^	29.4 ± 5.9 ^g^
F1	44.6 ± 5.3 ^cd^	0.56 ± 0.33 ^de^	18.0 ^f^± 2.4 ^f^	47.8 ± 2.8 ^f^
F2	58.7 ± 0.3 ^cd^	0.77 ± 0.22 ^cd^	31.3 ± 2.9 ^e^	52.0 ± 3.2 ^ef^
F3	63.7 ± 1.4 ^bc^	0.83 ± 0.06 ^c^	8.0 ± 4.3 ^g^	56.3 ± 1.3 ^de^
F4	74.3 ± 8.2 ^ab^	1.23 ± 0.14 ^b^	5.1 ± 3.1 ^g^	60.7 ± 1.1 ^d^
F5	89.8 ± 4.7 ^a^	1.62 ± 0.04 ^a^	64.0 ± 4.7 ^c^	197.8 ± 4.0 ^a^
F6	55.9 ± 5.1 ^de^	0.64 ± 0.29 ^cd^	58.0 ± 5.5 ^c^	97.0 ± 4.3 ^c^
Honey	31.5 ± 2.9 ^ef^	0.16 ± 0.11 ^f^	72.1 ± 1.8 ^b^	127.3 ± 4.6 ^b^
Hibiscus extract	57.7 ± 13.3 ^ab^	0.53 ± 0.18 ^e^	50.9 ± 2.6 ^d^	121.9 ± 1.0 ^b^
Strawberry concentrate	90.5 ± 20.2 ^a^	1.18 ± 0.24 ^b^	4.1 ± 3.3 ^g^	90.0 ± 8.3 ^c^

Results are mean ± standard deviation of triplicate analysis. Treatments with different letters within the same column are significantly different (*p* < 0.05).

**Table 4:** Average scores of prepared marshmallow sensory properties *.

Treatments *	Overall	Appearance	Softness	Lightness	Elasticity	Flavor
Control	5.806 ^c^	6.097 ^bc^	6.839 ^a^	6.355 ^ab^	6.452 ^ab^	5.774 ^bc^
F1	6.709 ^a^	7.097 ^a^	6.548 ^ab^	6.323 ^ab^	6.709 ^a^	7.258 ^a^
F2	5.839 ^bc^	5.645 ^c^	5.903 ^b^	5.613 ^b^	5.645 ^b^	5.032 ^c^
F3	6.226 ^abc^	6.290 ^abc^	6.581 ^ab^	6.452 ^ab^	6.387 ^ab^	7.484 ^a^
F4	6.968 ^a^	7.129 ^a^	6.484 ^ab^	6.806 ^a^	6.709 ^a^	7.032 ^a^
F5	6.613 ^abc^	6.581 ^ab^	6.677 ^ab^	6.709 ^a^	6.935 ^a^	6.774 ^ab^
F6	6.677 ^ab^	6.645 ^ab^	6.871 ^a^	6.742 ^a^	6.581 ^ab^	6.613 ^ab^

* Means of sensory attributes (overall, appearance, softness, lightness, elasticity, and flavor); results within the same column with different letters are significantly (*p* < 0.05) different.

**Table 5:** Nutritive value of the produced marshmallows and unit production cost (100 g).

	Control	F1	F2	F3	F4	F5	F6
Energy (Kcal)	296.0	330.1	286.8	261.5	237.9	231.1	331.6
Carbohydrate (g)	70.00	78.80	67.70	62.32	59.06	56.06	80.44
Lipid (g)	-	0.032	0.110	0.128	0.176	0.176	0.128
Protein (g)	4.000	5.456	5.536	5.568	5.628	5.658	5.588
Fiber (g)	-	0.018	0.722	0.894	1.336	1.356	0.884
Minerals (mg)							
Calcium	-	54.10	56.88	57.82	59.78	60.38	57.52
Iron	-	0.053	0.085	0.113	0.152	0.194	0.092
Magnesium	-	5.090	7.320	7.930	9.390	9.590	7.830
Phosphorous	-	38.74	42.28	43.30	45.66	40.06	43.10
Potassium	-	6.760	45.24	56.60	82.56	87.76	54.00
Sodium	-	0.420	5.270	6.580	9.740	10.14	6.380
Zinc	-	0.019	0.022	0.033	0.044	0.066	0.022
Vitamins							
Vitamin C (mg)	-	3.160	7.980	9.116	9.150	12.15	9.046
Thiamin (µg)	-	0.110	4.000	5.000	7.400	8.400	4.800
Riboflavin (µg)	-	7.500	8.000	10.80	15.00	18.80	14.40
Niacin (µg)	-	14.00	93.20	117.2	171.4	183.6	126.2
Vitamin B_6_ (µg)	-	2.200	2.400	3.600	4.800	7.200	6.000
Folate (mg)	-	0.1800	0.200	0.300	0.400	0.600	0.500
Vitamin B_12_ (µg)	-	-	-	-	-	-	-
Vitamin A (µg)	-	33.12	31.12	31.26	31.66	31.66	31.12
Vitamin D_3_ (µg)	-	0.480	0.480	0.480	0.480	0.480	0.480
Vitamin E (µg)	-	-	-	-	-	-	-
Cost (USD/100 g)	0.37	0.55	0.55	0.56	0.60	0.61	0.45

## Data Availability

The original contributions presented in this study are included in the article. Further inquiries can be directed to the corresponding author.
